# Comminuted lunate fracture combined with distal radius fracture and scaphoid fracture: A case report

**DOI:** 10.1097/MD.0000000000034393

**Published:** 2023-07-21

**Authors:** Jun Li, Guangyue Zhao, Weiliang Zhang

**Affiliations:** a Xi’an People’s Hospital, Shannxi, China.

**Keywords:** distal radius fracture, lunate fracture, polymethylmethacrylate cement, scaphoid fracture

## Abstract

Incidence of lunate fractures is very low, less than 1% of all fractures. Lunate fractures generally come from high-energy injuries, often combined with other wrist fractures. Simple lunate fractures can be fixed with screws or Kirschner wires. However, Comminuted lunate fractures are difficult to reduce and fixe by conventional methods.

**Patient concerns:**

Here we report a 42-year-old male construction worker who was crushed by an excavator bucket and presented with comminuted lunate fracture combined with distal radius fracture and scaphoid fracture.

**Diagnoses:**

Comminuted lunate fracture, distal radius fracture, and scaphoid fracture.

**Interventions:**

The posterior approach was used to reconstruct the radial lunate bone with polymethylmethacrylate cement, and cannulated screws were used to fix the scaphoid and distal radius fractures.

**Outcomes:**

At the 3rd month after surgery, the movement of the right wrist joint improved. At the sixth month after surgery, the patient returned to the building site and began working at the same intensity as before the injury.

**Lessons:**

Although the incidence of comminuted lunate fractures is very low, they occur sometimes. For comminuted lunate fractures, early identification and intervention can preserve most of the function of the wrist joint.

## 1. Introduction

Lunate fractures, especially comminuted lunate fractures are usually resulted from high energy trauma, and they are associated carpal injuries such as scaphoid, capitate, or radial styloid fractures in most cases. The exact incidence of lunate fracture has varied across studies, with the incidence ranging from only 0.5% to 6.5% of carpal fractures, and affecting males predominantly.^[1,2]^

Early diagnosis of lunate fractures are often difficult because Lunate bone is located in the center of carpal bones, which is easily affected by the overlapping of adjacent bones.^[3]^ Compared with simple lunate fracture, the early diagnosis of comminuted fracture is relatively easy because of its large morphological changes. On the other hand, compared with simple lunate fracture, comminuted lunate fracture has more blood supply destruction, higher incidence of lunate bone necrosis and greater difficulty in treatment.

To our knowledge, there are few reports of comminuted lunate fractures. We treated a case of comminuted lunate fracture with open reduction, internal fixation and bone cement reconstruction, and achieved satisfactory results. Herein, we report our methods and hope to provide ideas for the treatment of comminuted lunate fractures.

## 2. Case report

A 42-year-old male construction worker was admitted to our emergency department because of swelling, pain and restricted movement of the right wrist joint caused by mechanical extrusion. Symptoms began half an hour before our encounter, after the crushing on his right wrist by excavator bucket. He was diagnosed with right lunate comminuted fracture with distal radius fracture and scaphoid fracture by the radiography combined with physical signs and symptoms. He was in good health in the past, and had no other history of trauma except that the right wrist joint was squeezed this time.

On admission, physical examination showed swelling, malformation, extensive tenderness, percussion pain and bone friction sign of the right wrist. The flexion, extension, ulnar deviation and radial deviation of the right wrist joint were significantly limited. The flexion of the right metacarpophalangeal joint was limited, and the activities of interphalangeal joint and elbow joint were normal. The skin sensation and blood circulation of the right hand were normal.

**Figure 1.**
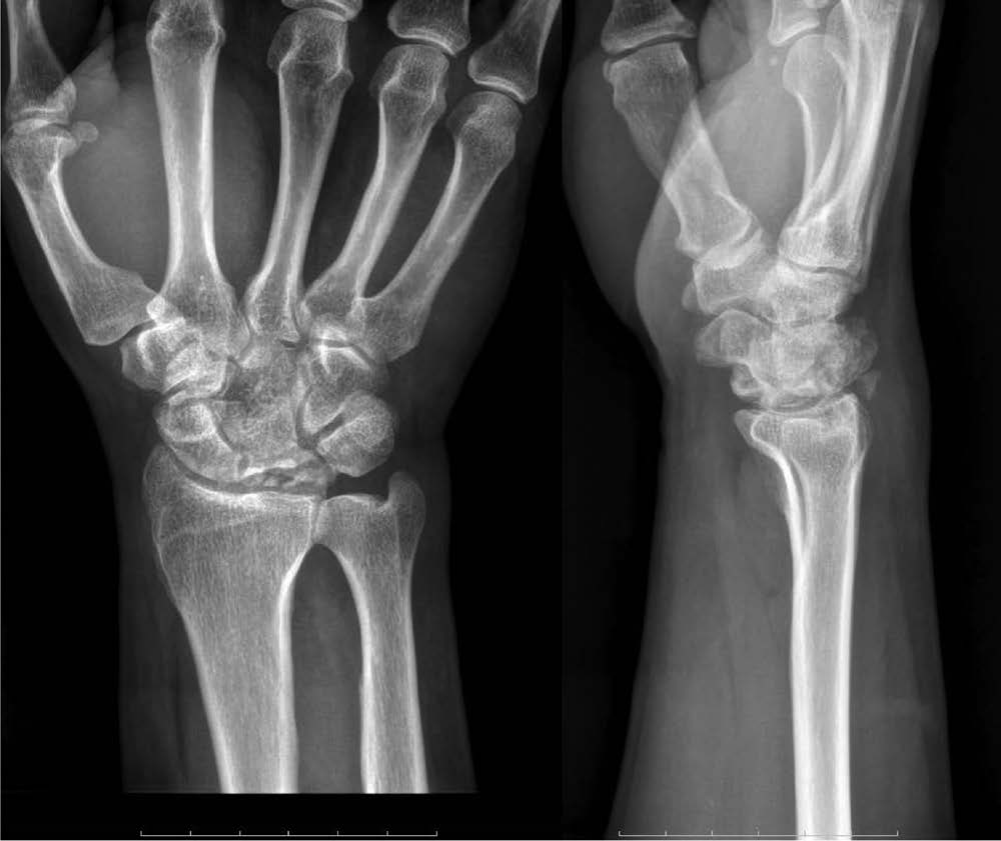
Preoperative radiograph showing abnormal morphology of the right lunate bone, and patchy high-density shadows beside the triangular bone.

**Figure 2.**
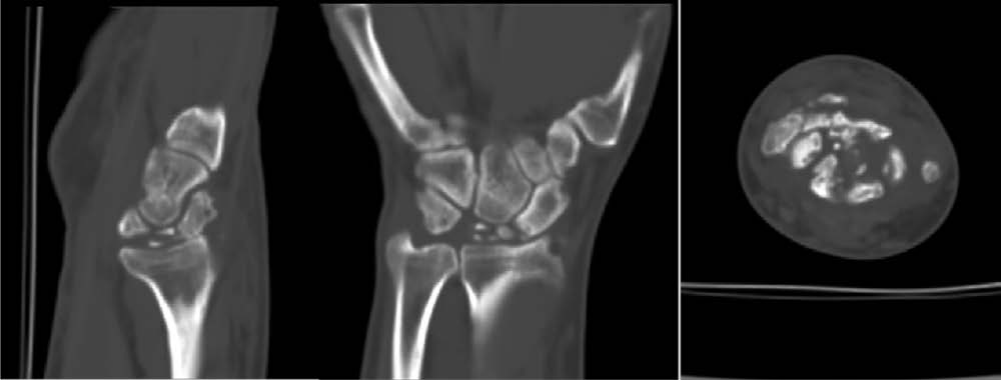
Computed tomography showing a comminuted fracture of the right lunate, a transverse fracture line in the scaphoid and styloid process of the radius.

The anteroposterior and lateral X-ray films of the right wrist showed abnormal morphology of the right lunate bone, with multiple transparent linear shadows, and patchy high-density shadows beside the triangular bone (Fig. 1). Three dimensional computed tomography of the right wrist joint showed a comminuted fracture of the right lunate, a transverse fracture line in the scaphoid and styloid process of the radius (Fig. 2).

**Figure 3.**
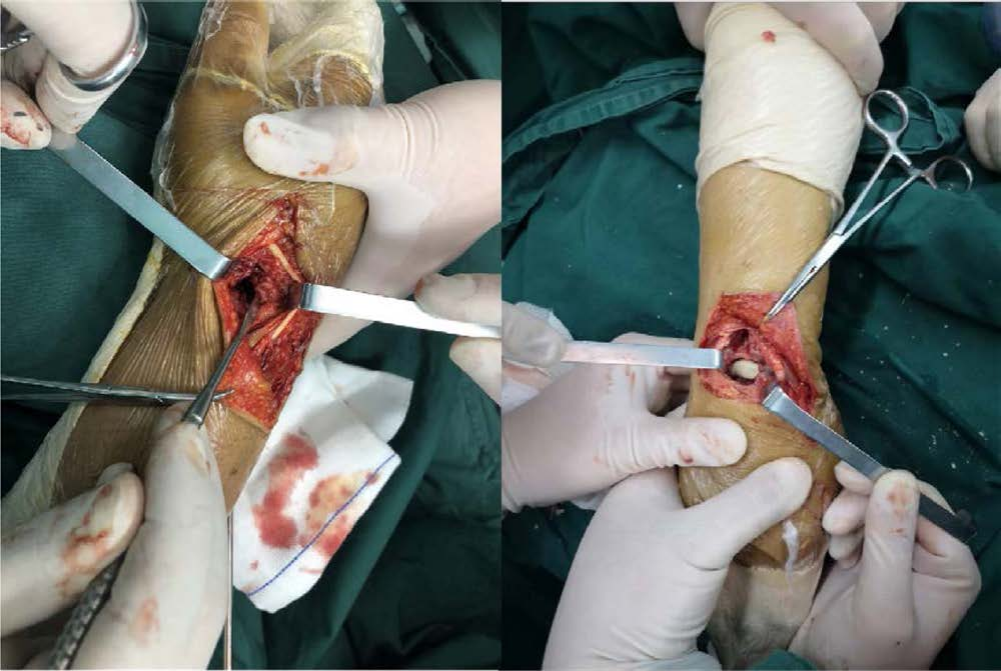
After removing the fragments of lunate bone, the lunate bone was reconstructed with PMMA and replanted in situ. PMMA = polymethylmethacrylate cement.

**Figure 4.**
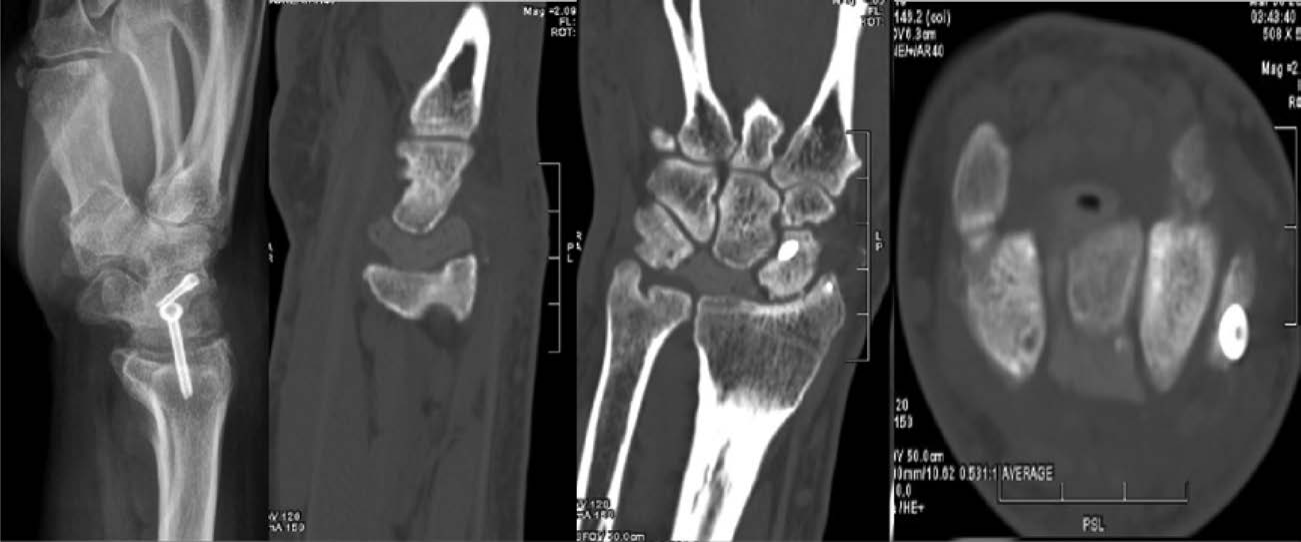
Distal radius fractures and scaphoid fractures healed well, and the position of the lunate prosthesis is satisfactory (3 mo after surgery).

On the fifth day after the injury, he underwent lunate bone reconstruction and open reduction and internal fixation of distal radius and scaphoid fractures under brachial plexus block anesthesia. We chose the dorsal approach of wrist joint. Firstly, the fractures of scaphoid and distal radius were fixed with cannulated screws. Secondly, the lunate bone was exposed between the extensor digitorum tendon and extensor digitorum tendon. After removing the fragments of lunate bone, the lunate bone was reconstructed with polymethylmethacrylate cement (PMMA) and replanted in situ (Fig. 3). Postoperative X-ray and computed tomography examination showed well reduction and fixation of distal radius and scaphoid fractures, and the lunate prosthesis was in a satisfactory position (Fig. 4). After the operation, the wrist joint was fixed with plaster for 4 weeks, and then functional exercise started.

**Figure 5.**
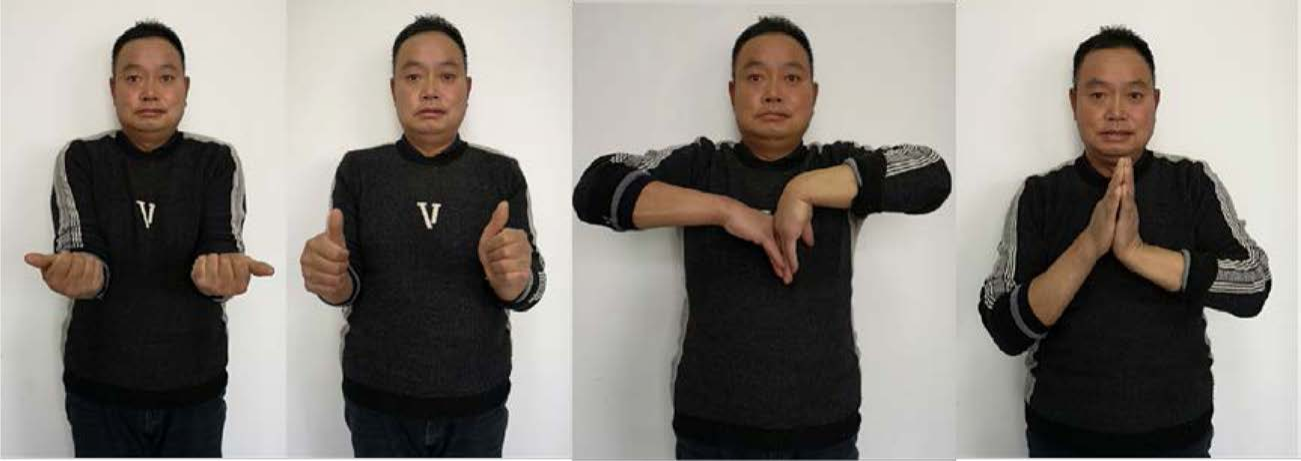
The patient’s wrist function has basically recovered (6 mo after surgery).

At the 3rd month after surgery, he presented with normal pronation and supination, ulnar deviation and radial deviation of the right wrist, but slight limitations in flexion and back extension activities compared to the left wrist (Fig. 5). He was radiologically reexamined at 3rd month after surgery, and the fracture in the right wrist healed well, and the shape of the lunate prosthesis was good, there was no sign of dislocation, and there was no arthritis in the adjacent carpal bones. In the sixth month after surgery, he returned to the building site and began working at the same intensity as before the injury. During the 1-year follow-up, there was no difference in function between the right wrist joint and the left wrist joint, and there was no swelling or pain as well.

## 3. Discussion

The lunate bone is located between the navicular bone and the triangle, and is jointed with the cephaloid bone and the uncinate bone respectively.^[4]^ In the process of trauma, the lunate bone rotates to a certain extent on the articular surface of the lower radius and the capitate bone, which can eliminate some external forces. So the incidence of lunate fracture, especially comminuted lunate fracture, is very low.

In 1921, Roth reported the first case of lunate fracture.^[5]^ Since then, more and more cases of lunate fracture have been reported, but there are few cases of comminuted lunate fracture. To our best knowledge, this is the first case report on comminuted lunate fracture combined with distal radius fracture and scaphoid fracture.

Lunate fracture usually occurs at the same time as other wrist fractures or dislocations, and may occur alone in a few cases.^[6]^ In most cases, lunate fracture mainly comes from indirect violence, and often occurs in the axial impact of radius in the state of dorsal extension of wrist joint. Comminuted fracture of lunate bone often comes from direct violence. For example, in this case, the excavator bucket directly squeezed the lunate bone, and the wrist joint was forced ulnar deviation and the pressure was concentrated on the lunate bone from multiple directions during the squeezing process, resulted in comminuted fracture of lunate bone. At the same time, ulnar deviation and intercarpal supination drive the scaphoid outward away from the lunate, resulted in the fracture of the distal radius and the styloid process of the radius.

Teisen–Hjarbaek classified lunate bone fractures into 5 groups according to their radiological appearances and according to the vascular anatomy of the Iunate.^[2]^ Our reported case is very rare and the lunate bone was shattered into numerous fragments, it does not belong to any type of Teisen—Hjarbaek classification.

The blood supply of the lunate bone comes from the arterial network of the carpometacarpal and dorsal sides, mainly from the palmar and medial arteries of the carpometacarpal branch of the radial artery. According to the way in which the nutrient artery enters the lunate bone, Gelberman and Gross^[7]^ divided the blood supply of the lunate bone into 3 types: type X, type Y and type I. And fractures which damaged the nutrient artery may lead to aseptic necrosis. In this case, the comminuted lunate fracture resulted in severe intraosseous destruction of the nutrient vessels, and there was a great risk of lunate bone necrosis.

There is no consensus on the treatment of lunate fracture. It is generally believed that early intervention is of great significance for the prevention of aseptic necrosis of lunate bone.^[8]^ For lunate fracture without obvious displacement, external fixation can achieve a satisfactory therapeutic effect in most cases. Surgical intervention is often necessary for patients with obvious fracture displacement, lunate dislocation or multiple fractures and dislocations of the wrist.^[9]^ Kirschner wire or screw fixation is a common treatment for lunate fracture.^[10,11]^ For the cases of comminuted lunate fracture, on the 1 hand, the risk of aseptic necrosis is high due to the destruction of blood supply; On the other hand, the traditional treatment can not meet the requirements of reduction and fixation, which is a challenging problem with no proven treatment. According to literatures, metacarpal longus muscle reconstruction or metal prosthesis reconstruction after lunate bone resection has achieved satisfactory results in the treatment of Kienbock’s disease.^[12,13]^ Referring to the treatment of Kienbock’s disease, we finally chose PMMA reconstruction after lunate bone resection to treat the comminuted lunate bone fracture. Three months later, the wrist function was completely recovered, and there was no swelling or pain of the wrist in 1-year’s follow-up.

It should be noted that the follow-up time of our cases is only 1 year. The effection of PMMA reconstruction in the treatment of comminuted lunate fractures needs to be further verified by more cases and longer follow-up time.

In conclusion, in the absence of a better treatment, PMMA reconstruction can be used as an effective treatment for refractory lunate fracture.

## Author contributions

**Conceptualization:** Guangyue Zhao.

**Investigation:** Weiliang Zhang.

**Methodology:** Jun Li, Guangyue Zhao.

**Resources:** Jun Li, Weiliang Zhang.

**Supervision:** Guangyue Zhao, Weiliang Zhang.

**Validation:** Weiliang Zhang.

**Visualization:** Guangyue Zhao, Weiliang Zhang.

**Writing – original draft:** Jun Li, Guangyue Zhao, Weiliang Zhang.

**Writing – review & editing:** Jun Li, Guangyue Zhao, Weiliang Zhang.
